# Antibacterial Activity of *Chrysanthemum buds* Crude Extract Against *Cronobacter sakazakii* and Its Application as a Natural Disinfectant

**DOI:** 10.3389/fmicb.2020.632177

**Published:** 2021-02-03

**Authors:** Yunhe Chang, Min Xing, Xinying Hu, Hongxia Feng, Yao Wang, Bingrui Guo, Maocheng Sun, Lizhi Ma, Peng Fei

**Affiliations:** ^1^Food and Pharmaceutical Engineering Institute, Guiyang University, Guiyang, China; ^2^Guizhou Fruit Processing Engineering Technology Research Center, Guiyang, China; ^3^College of Food and Bioengineering, Henan University of Science and Technology, Luoyang, China; ^4^College of Food Science and Engineering, Changchun University, Changchun, China

**Keywords:** *Cronobacter sakazakii*, *Chrysanthemum buds* crude extract, antibacterial activity, mechanism, application

## Abstract

*Cronobacter sakazakii* is an opportunistic food-borne pathogen that endangers the health of neonates and infants. This study aims to elucidate the antibacterial activity and mechanism of *Chrysanthemum buds* crude extract (CBCE) against *C. sakazakii* and its application as a natural disinfectant. The antibacterial activity was evaluated by the determination of the diameter of inhibition zone (DIZ), minimum inhibitory concentration (MIC), and minimum bactericide concentration (MBC). The antibacterial mechanism was explored based on the changes of growth curve assay, intracellular ATP concentration, membrane potential, intracellular pH (pH_in_), content of soluble protein and nucleic acid, and cell morphology. Finally, the inactivation effects of CBCE against *C. sakazakii* in biofilm on stainless steel tube, tinplate, glass, and polystyrene were evaluated. The results showed that the DIZ, MIC, and MBC of CBCE against *C. sakazakii* were 14.55 ± 0.44–14.84 ± 0.38 mm, 10 mg/mL, and 20 mg/mL, respectively. In the process of CBCE acting on *C. sakazakii*, the logarithmic growth phase of the tested bacteria disappeared, and the concentrations of intracellular ATP, pH_in_, bacterial protein, and nucleic acid were reduced. Meanwhile, CBCE caused the cell membrane depolarization and leakage of cytoplasm of *C. sakazakii*. In addition, about 6.5 log CFU/mL of viable *C. sakazakii* in biofilm on stainless steel tube, tinplate, glass, and polystyrene could be inactivated after treatment with 1 MIC of CBCE for 30 min at 25°C. These findings reveal the antibacterial activity and mechanism of CBCE against *C. sakazakii* and provide a possibility of using a natural disinfectant to kill *C. sakazakii* in the production environment, packaging materials, and utensils.

## Introduction

*Cronobacter sakazakii*, a gram-negative, facultatively anaerobic, motile emerging food-borne pathogen, can adhere tightly to the surface of equipment, packaging materials, and utensils due to its strong ability for biofilm formation, which is believed to be one of the important reasons for the contamination of food by this pathogen (Friedemann, 2007; Amalaradjou and Venkitanarayanan, 2011). Importantly, *C. sakazakii* can cause some serious life-threatening diseases including sepsis, meningitis, and necrotizing enterocolitis in infants and adults with immunodeficiency, with a 40–80% mortality rate (Hunter and Bean, 2013). In 2002, the International Commission on Microbiological Standards for Foods (ICMSF) defined *C. sakazakii* as “a pathogen that leads to serious damage, endangers life or causes long-term, chronic and substantial sequelae to the specific population” (Iversen and Forsythe, 2003). Therefore, it is significant to prevent and control *C. sakazakii* for the guarantee of food safety and public health.

Previous studies have shown that *C. sakazakii* mainly leads to food safety risks by contaminating processing equipment, packaging materials, and utensils (Kusumaningrum et al., 2003; Brandl et al., 2014). Currently, the most common way to eliminate *C. sakazakii* from the environment in which food is exposed is still the use of chemical disinfectants (Meireles et al., 2016). However, *C. sakazakii* strains have strong efficient biofilm formation ability, which helps weaken the bactericidal effect of chemical disinfectants (Lehner et al., 2005; Kim et al., 2006). Meanwhile, due to the long and unscientific use of chemical disinfectants, some *C. sakazakii* strains resistant to chemical disinfectants have been found (Park et al., 2015). In addition, the potential toxicity and environmental unfriendliness of chemical disinfectants cannot be ignored, which make consumers unwilling to accept them (Meireles et al., 2016). Therefore, a natural disinfectant with good antibacterial effect is urgently needed.

In recent years, a growing number of researchers are focusing their attention on screening natural antibacterial extracts to inhibit food-borne pathogens, because of their safety and nature (Fei et al., 2018a; Guo L. et al., 2020). *Chrysanthemum morifolium* is a traditional medicated and edible cash crop and contains polyphenols, flavones, essential oils, and other chemical components, which have been shown to have strong antioxidant, anti-inflammatory, and antibacterial effects (Kim and Lee, 2005; Cui et al., 2014; Kuang et al., 2018). *Chrysanthemum buds* is made by drying the *C. morifolium* flowers when they are not fully opened and is widely used in tea drinks and traditional Chinese medicine for its stronger anti-inflammatory effect in China, suggesting that *C. buds* extract should be used in the development of natural disinfectants.

With this background, the purpose of this study was to assess the antibacterial activity of *C. buds* crude extract (CBCE) against *C. sakazakii* and explore the possible action approach by revealing the changes in the growth curves, intracellular ATP concentration, cell membrane potential, intracellular pH (pH_in_), levels of bacterial protein and nucleic acid, and cell morphology. Moreover, its application as a natural disinfectant was implemented and evaluated.

## Materials and Methods

### Experimental Materials

All growth media were obtained from Qingdao Hopebio Biotechnology Co. Ltd. (Qingdao, China). All reagents were purchased from Shanghai MacLean Biochemical Technology Co., Ltd. (Shanghai, China). The stainless steel tube, tinplate, glass, and polystyrene were supplied by Luoyang Zhenguo Packaging Material Co. Ltd. (Luoyang, China).

### Bacterial Strains

A total of 8 *C. sakazakii* strains were used in the study; among them, *C. sakazakii* ATCC 29544 was provided from the American Type Culture Collection (ATCC, Manassas, VA, United States), and *C. sakazakii* PD2, IN3, CFP6, FP1, V1, F2, and C1 were isolated from prepared dishes, instant noodles, chilled fresh pork, PIF, vegetables, fruits, and cookies, respectively, which has been reported in our previous study (Fei et al., 2018b). All strains were used to evaluate the antibacterial activity of CBCE, and *C. sakazakii* ATCC 29544 was used to analyze the antibacterial mechanism. Before the experiment was carried out, all strains were stored in Luria–Bertani (LB) broth with 20% glycerol (v/v) at −80°C. The above cultures were streaked on tryptone soya agar (TSA) and incubated at 37°C for 24 h. A single colony of each strain was inoculated into LB broth, followed by incubation at 37°C for 12 h for subsequent study.

### Preparation of CBCE

The *C. buds* were crushed and passed through an 82-mesh sieve to obtain their powders. These powders were dissolved in sterile distilled water (SDW) with a ratio of 1:30 (m:v) and extracted at 70°C for 80 min. Then, the filtrate was collected and the filter residues were further extracted twice by the same method. All the filtrate was concentrated using a rotary evaporator (Zhejiang Nader Scientific Instrument Co. Ltd., Hangzhou, China) at 40°C for 10 h to obtain the paste of CBCE. Finally, the paste was dried into powder by a vacuum freeze-drying machine (Tokyo Riken Co., Ltd., Tokyo, Japan). Specific parameters are as follows: paste thickness is 3 mm, pre-cooling temperature is −55°C, vacuum pressure is 45 Pa, sublimation temperature is 40°C, and drying time is 16 h. The images of *C. buds* and CBCE powders are shown in Supplementary Figure 1, and the main chemical components of CBCE are shown in Supplementary Table 1.

### Determination of the DIZ

The diameter of inhibition zone (DIZ) of CBCE against *C. sakazakii* used the Oxford cup method according to the previous report (Xu et al., 2019). Briefly, 100 μL of *C. sakazakii* bacterial suspensions (about 10^7^ CFU/mL) was uniformly plated on the sterilized TSA plates. Three sterile oxford cups with a diameter of 8 mm (Shanghai Precision Instrument Co., Ltd., Shanghai, China) were evenly placed on the TSA surface and gently pressed with a tweezer. Then, 200 μL of CBCE (10 mg/mL) was added in the oxford cups, followed by incubation at 37°C for 24 h. Finally, the DIZ value was determined by taking an average of the diameters of three transparent inhibition zones on the same plate.

### Determination of MIC and MBC

The minimum inhibitory concentration (MIC) and minimum bactericide concentration (MBC) of CBCE against eight *C. sakazakii* strains were determined using the agar dilution method as previously described (Fei et al., 2018a). In brief, 500 μL of TSA media containing different concentrations of CBCE (0, 0.3125, 0.625, 1.25, 2.5, 5, 10, and 20 mg/mL) was added into sterile 24-well plates (Shanghai Precision Instrument Co., Ltd., Shanghai, China), respectively. After the TSA medium was solidified, 2 μL (10^6^ CFU/mL) of bacterial suspension was uniformly coated on the surface of agar block followed by incubation at 37°C for 24 h. MIC was defined as the lowest concentration at which CBCE completely prevented *C. sakazakii* from growing under visual observation. Further, 100 μL (10^6^ CFU/mL) of *C. sakazakii* was treated with 10, 20, 40, and 80 mg/mL of CBCE for 30 min, respectively, then was spread onto TSA plates and incubated at 37°C for 24 h. The lowest concentration at which no colonies were observed was considered as the MBC of CBCE against *C. sakazakii*. In addition, the TSA medium without CBCE was used as negative control, and the TSA medium containing 0.1 mg/mL ampicillin was used as positive control.

### Growth Curves

The growth curves of *C. sakazakii* ATCC 29544 strains with different treatments were determined according to the method described by Shi et al. (2016a), with minor modifications. One milliliter of tested bacteria cultures (OD_600_ = 0.1) was added into the 96-well microtiter plates (Shanghai Precision Instrument Co., Ltd., Shanghai, China), then CBCE was added to the above solution and mixed well, and the final concentration was adjusted to 0 (control), 0.5, 1, 1.5, and 2 MIC. All samples were cultured at 37°C for 24 h, the optical density (OD)_600_ values of which were measured by a multimode plate reader (Tecan, Infinite M200 PRO, Männedorf, Switzerland) every 2 h to assess bacterial growth.

### Measurement of Intracellular ATP Concentrations

After 10^7^ CFU/mL of *C. sakazakii* ATCC 29544 strains were treated by 0, 1, and 2 MIC of CBCE at 37°C for 30 min, the intracellular ATP concentrations of tested bacteria were determined on the basis of the previous report (Sánchez et al., 2010). The treated strains were cracked using the ultrasound treatment, and then a treatment of 100°C for 3 min was performed to inactivate the ATP in the system. The above samples were centrifuged at 8,000 × *g* for 3 min at 37°C to obtain the detected supernatant. Finally, the relative luminescence value representing the intracellular ATP concentration of the tested bacteria was determined using an ATP kit (AmyJet Scientific Inc., Wuhan, China) according to the product instructions.

### Membrane Potential Determination

The method reported by Guo et al. (2019) was followed to determine the membrane potential of the tested bacteria. Briefly, 125 μL of bacterial suspension (10^7^ CFU/mL) and 0.5 μL of bis-(1,3-dibutylbarbituriis-(1,3-dibutylbarbituric acid) trimethine oxonol (Beijing Solarbio Science and Technology Co. Ltd., Beijing, China) fluorescent probe were added into a black and opaque 96-well plate and cultured at 37°C for 30 min. Then, CBCE was added to the 96-well plate, and the final concentrations were adjusted to 1 and 2 MIC, respectively. The untreated bacterial suspension was used as a control. After incubating at 37°C for 30 min, the fluorescence intensities of all samples at the excitation wavelength and emission wavelength of 492 and 515 nm, respectively were measured using a multifunctional microplate reader (Beijing Potenov Technology Co. Ltd., Beijing, China).

### Determination of pH_in_

The effects of CBCE on pH_in_ of *C. sakazakii* ATCC 29544 strains were analyzed based on the method as described by Guo L. et al. (2020). In brief, 1.0 μM carboxyfluorescein diacetate succinimidyl ester dye as the fluorescent probe was added to 10^8^ CFU/mL of bacterial suspensions, then incubated at 37°C for 30 min. After a centrifugation at 11200 × *g* for 5 min, the cell pellets were obtained and suspended into sterilized normal saline (NS) followed by addition of glucose (10 mM). The mixed system was incubated at 37°C for 30 min and centrifuged at 11,200 × *g* for 5 min to get the cell pellets. Then, these cell pellets were washed twice and resuspended in NS. One hundred and twenty-five microliters of bacterial suspension was treated by 0, 1, and 2 MIC of CBCE in a black and opaque 96-well plate at 37°C for 20 min. The fluorescence intensities were detected using a multimode plate reader (Tecan, Infinite M200 PRO, Männedorf, Switzerland) at 25°C under the excitation wavelength of 440 nm and emission wavelength of 490 nm. The ratio of fluorescence intensities at 490/440 nm was considered as the pH_in_ of cells. In addition, the fluorescence intensity of the cell-free control was deducted to eliminate the background error.

### Measurement of *C. sakazakii* Protein Levels

About 10^7^ CFU/mL of *C. sakazakii* ATCC 29544 suspension was treated with 1 MIC of CBCE at 37°C for 3, 6, 9, and 12 h, respectively. The untreated *C. sakazakii* ATCC 29544 was used as a control. All samples were centrifuged at 11,200 × *g* for 5 min to obtain the cell pellets and supernatants. The pellets were resuspended in NS and analyzed by sodium dodecyl sulfate-polyacrylamide gel electrophoresis (SDS-PAGE) to determine the effect of CBCE on protein in bacteria according to our previous study (Fei et al., 2018a). The protein contents in supernatants were analyzed to determine the amount of protein leaking by the Coomassie bright blue method as described by He et al. (2014).

### Measurement of Nucleic Acid Leakage

The method reported by Ma et al. (2016) was performed to determine the nucleic acid content of *C. sakazakii* ATCC 29544. Approximately 10^9^ CFU/mL of bacterial suspensions was treated with 0, 0.5, 1, 1.5, and 2 MIC of CBCE, respectively, then incubated at 37°C for 12 h. Every 2 h during incubation, 200 μL of cultures was absorbed and centrifuged at 11,200 × *g* for 10 min to obtain the supernatants. The OD_260_ values of supernatants were measured by a multimode plate reader (Tecan, Infinite M200 PRO, Männedorf, Switzerland).

### Transmission Electron Microscope Observation

The effects of CBCE on the cell morphology of *C. sakazakii* ATCC 29544 were observed using a transmission electron microscope (TEM) according to the previous report (Fei et al., 2019). Briefly, *C. sakazakii* strains were treated by 0 (control), 1, and 2 MIC of CBCE for 4 h followed by centrifuging at 3,000 × *g* for 5 min to collect the cell pellets. The cell pellets were fixed with 0.1 M sodium phosphate buffer containing 2.5% glutaraldehyde for 12 h, then washed three times with NS. Further, the cells were stored in 1% osmium tetroxide at room temperature for 6 h followed by washing three times with NS again. After the above treatments, the cells were dehydrated with 50, 70, 90, and 100% ethanol for 10 min and embedded in Epon Lx-112 (Ladd Research, Williston, VT, United States). Finally, the samples were stained with uranyl acetate and lead citrate, then observed under TEM (Hitachi, Tokyo, Japan).

### Application of CBCE as a Natural Disinfectant

The inhibitory abilities of CBCE against *C. sakazakii* ATCC 29544 in biofilm on stainless steel tube, tinplate, glass, and polystyrene were assessed according to the report of Fei et al. (2020). In brief, all the materials were sterilized and cut into 50 mm × 20 mm coupons then placed into 50-mL tubes containing 30 mL of bacterial suspensions (10^7^ CFU/mL) followed by incubation at 4°C for 24 h. The coupons were washed twice with SDW for 10 s and cultured in 30 mL of LB broth at 25°C for 24 h then washed twice with SDW for 10 s. The samples were treated with 0, 1, and 2 MIC of CBCE and collected at 0, 10, 20, and 30 min, respectively, then washed twice with 300 mL of SDW for 10 s. Then, 30 mL of sterile phosphate-buffered saline and 3 g glass bead (300–600 μm) were added into the tubes containing the coupon, followed by swirling for 5 min. Finally, the plate count method was used to determine the viable counts of *C. sakazakii* in phosphate-buffered saline (PBS, pH 7.2). The PBS suspension was serially diluted and plated onto TSA plates, followed by incubation at 37°C for 24 h to enumerate the viable count of *C. sakazakii*.

### Statistical Analysis

All experiments except SDS-PAGE and TEM analysis were repeated three times. The data were expressed as mean values ± standard deviation (SD). The significance test was performed by analysis of variance (ANOVA) in the SPSS 20.0 software (SPSS Inc., Chicago, IL, United States). *P* < 0.05 was considered as significant difference.

## Results

### DIZ, MIC, and MBC of CBCE Against *C. sakazakii*

The DIZs, MICs, and MBCs of CBCE against eight *C. sakazakii* strains are given in Table 1. The results showed that the DIZs ranged from 14.55 ± 0.44 to 14.84 ± 0.38 mm, and no significant difference (*P* > 0.05) was found between these strains. The MICs and MBCs of CBCE against all *C. sakazakii* strains were 10 and 20 mg/mL, respectively.

**TABLE 1:** Antimicrobial activity of CBCE against all eight *C. sakazakii* strains.

*C. sakazakii* strains	Source	DIZ (mm)	MIC (mg/mL)	MBC (mg/mL)
ATCC 29544	American Type Culture Collection	14.63 ± 0.25^a^	10	20
PD2	Prepared dishes	14.55 ± 0.44^a^	10	20
IN13	Instant noodles	14.67 ± 0.23^a^	10	20
CFP6	Chilled fresh pork	14.84 ± 0.38^a^	10	20
FP1	PIF	14.67 ± 0.44^a^	10	20
V1	Vegetables	14.56 ± 0.32^a^	10	20
F2	Fruits	14.74 ± 0.36^a^	10	20
C1	Cookies	14.61 ± 0.47^a^	10	20

*Values are means ± SD from triplicate determinations.*

*Columns values with different letters were significantly different (*P* < 0.05).*

### Growth Curves

**FIGURE 1:**
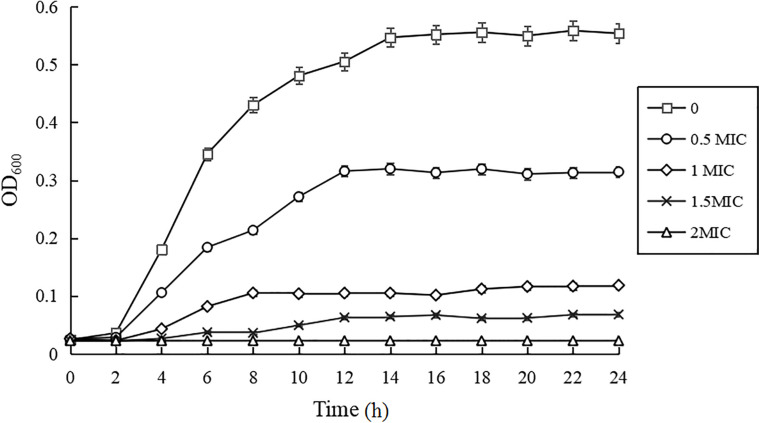
Growth curves of *C. sakazakii* ATCC 29544 treated with various concentrations of CBCE. Bars represent the SD (*n* = 3).

As shown in Figure 1, compared with the control group, the growth rates of *C. sakazakii* ATCC 29544 treated by CBCE were obviously reduced and slowed down with the increase of CBCE concentration. When *C. sakazakii* was treated by 1 MIC of CBCE, the logarithmic phase of *C. sakazakii* disappeared. When treating by 2 MIC, *C. sakazakii* stagnated in the delayed stage and stopped growing.

### Changes in Intracellular ATP Concentration

**FIGURE 2:**
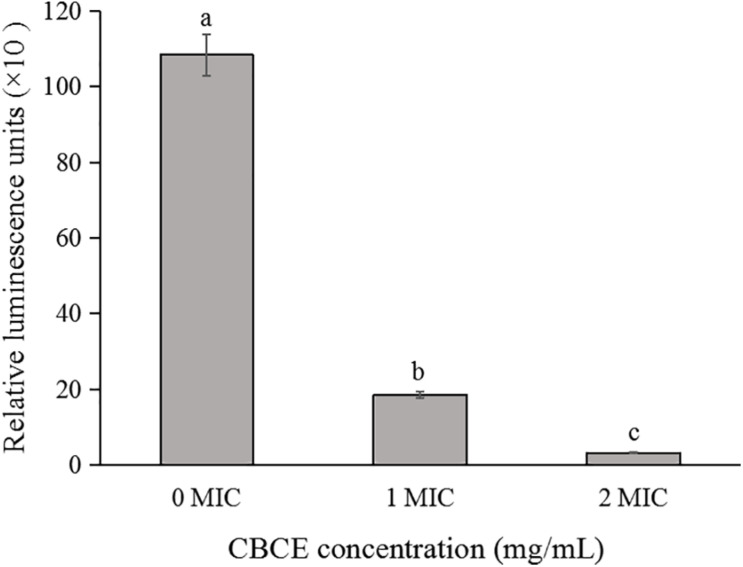
Effects of CBCE on the intracellular ATP concentrations of *C. sakazakii* ATCC 29544. The mean values of the three independent experiments were used for statistical analysis. Bars represent the SD (*n* = 3). Different letters represent significant differences (*P* < 0.05).

The effect of CBCE on the intracellular ATP concentration of *C. sakazakii* ATCC 29544 is shown in Figure 2. Compared to the control group, the intracellular ATP concentration of *C. sakazakii* ATCC after treatments with 1 and 2 MIC of CBCE decreased significantly (*P* < 0.05) with the increase of concentration.

### Changes in Membrane Potential

**FIGURE 3:**
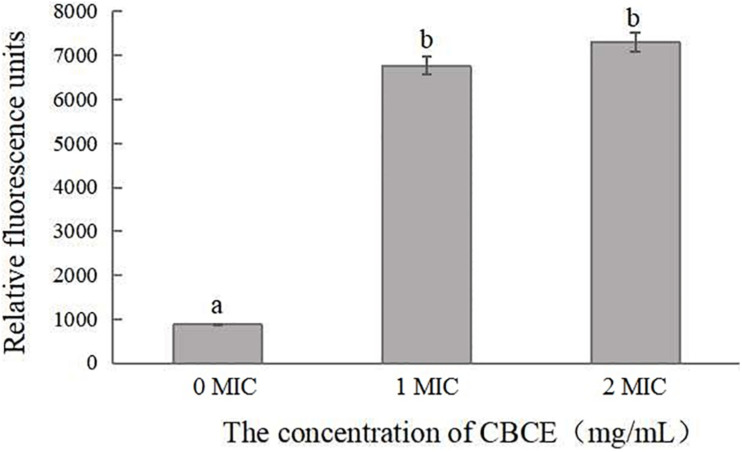
Effects of CBCE on the membrane potential of *C. sakazakii* ATCC 29544. The mean values of the three independent experiments were used for statistical analysis. Bars represent the SD (*n* = 3). Different letters represent significant differences (*P* < 0.05).

Figure 3 illustrates the changes in membrane potential of *C. sakazakii* ATCC 29544 after treatments with 0, 1, and 2 MIC of CBCE. It can be seen that the cell membrane potential of experimental groups was significantly increased in comparison with the control (*P* < 0.05), indicating that the membrane potential depolarization occurred in cells treated with CBCE.

### Changes in pH_in_

**FIGURE 4:**
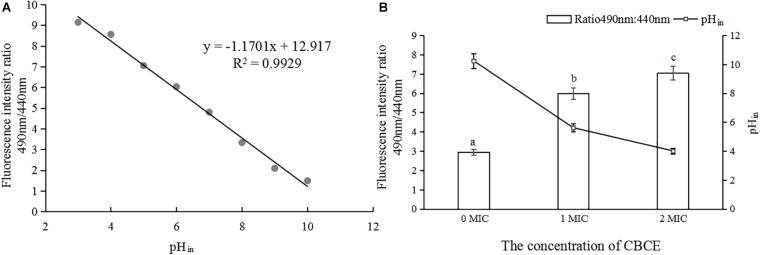
Effects of CBCE on the pH_in_ of *C. sakazakii* ATCC 29544. **(A)** The linear relationship between pH_in_ and the ratio of fluorescence intensity at 490 and 440 nm. **(B)** Changes in pH_in_ of *C. sakazakii* ATCC 29544 after treatments with 1 and 2 MIC of ATCE. The mean values of the three independent experiments were used for statistical analysis. Bars represent the SD (*n* = 3). Different letters represent significant differences (*P* < 0.05).

As shown in Figure 4A, the pH_in_ value had a good linear relationship with the fluorescence ratio of 490 nm and 440 nm (*y* = −1.1701*x* + 12.917, *R*^2^ = 0.9929). Figure 4B shows that with the addition of CBCE, the pH_in_ value of *C. sakazakii* ATCC 29544 decreased significantly, and there were significant differences among the three groups (*P* < 0.05). Specifically, after treatments with 1 and 2 MIC of CBCE, the pH_in_ value dropped from 7.68 ± 0.52 to 4.21 ± 0.19 and 3.01 ± 0.10, respectively.

### Changes in *C. sakazakii* Protein Levels

**FIGURE 5:**
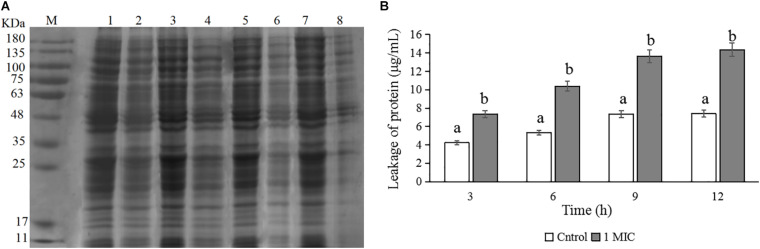
Effects of CBCE on the protein level of *C. sakazakii* ATCC 29544. **(A)** SDS-PAGE analysis of the protein in cells treated with 0 and 1 MIC of CBCE. Lane M: marker; Lanes 1, 3, 5, and 7: untreated for 3, 6, 9, and 12 h; Lanes 2, 4, 6, and 8: treated with 1 MIC of CBCE for 3, 6, 9, and 12 h. **(B)** The amount of protein leaking from cells treated with 0 and 1 MIC of CBCE for 3, 6, 9, and 12 h. The mean values of the three independent experiments were used for statistical analysis. Bars represent the SD (*n* = 3). Different letters represent significant differences (*P* < 0.05).

The changes in protein levels of *C. sakazakii* ATCC 29544 after treatment with 1 MIC of CBCE are presented in Figure 5. The SDS-PAGE image (Figure 5A) showed the changes in the bacterial protein where the protein bands were obviously weaker and almost disappeared when 1 MIC of CBCE was applied for 12 h. The Coomassie bright blue test result (Figure 5B) showed that the amount of protein leaking from the bacteria significantly increased at different treatment times.

### Leakage of the Nucleic Acid

**FIGURE 6:**
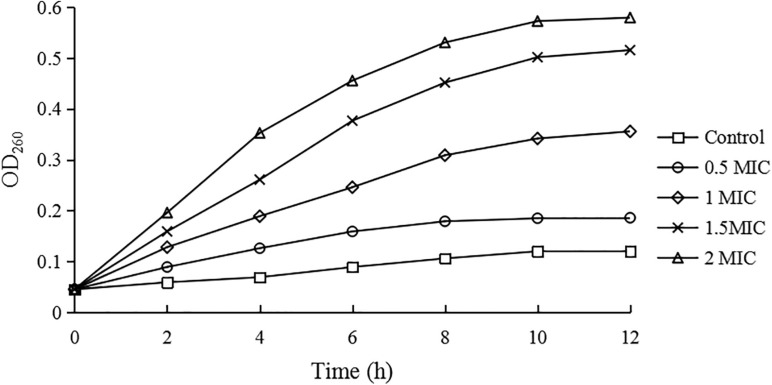
The nucleic acid leakage of *C. sakazakii* ATCC 29544 after treatments with different concentrations of CBCE. Bars represent the SD (*n* = 3).

The amounts of nucleic acid leakage from *C. sakazakii* ATCC 29544 cells were expressed as the absorbance value at 260 nm and given in Figure 6. After treatments with CBCE, the OD_260 nm_ values of supernatants increased significantly with the increase of concentrations, suggesting that CBCE could cause a large amount of nucleic acid leakage from the tested cells.

### TEM Observation

**FIGURE 7:**
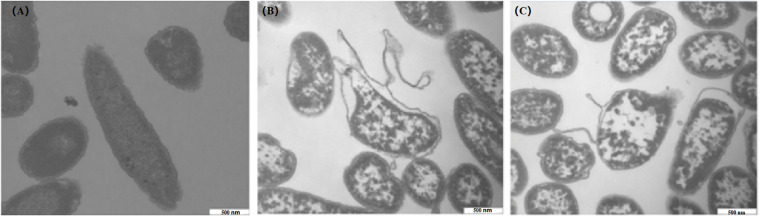
TEM observations (40,000 × magnification) of *C. sakazakii* ATCC 29544 cells after different treatments. **(A)** Untreated for 4 h, **(B)** treated with 1 MIC of CBCE for 4 h, and **(C)** treated with 2 MIC of CBCE for 4 h.

After treatments with 1 and 2 MIC of CBCE for 4 h, TEM was used to observe the changes in *C. sakazakii* ATCC 29544 cell morphology and is shown in Figure 7. The untreated cell was used as the control (Figure 7A), which showed regular morphology, smooth surface, and uniform cytoplasm. After treatments with CBCE, the deformation, collapse, and leakage of cell fluid occurred in the tested cells, and the damage degree of cell morphology was more serious with the increase of CBCE concentration (Figures 7B,C).

### Inactivation Activity of CBCE

**FIGURE 8:**
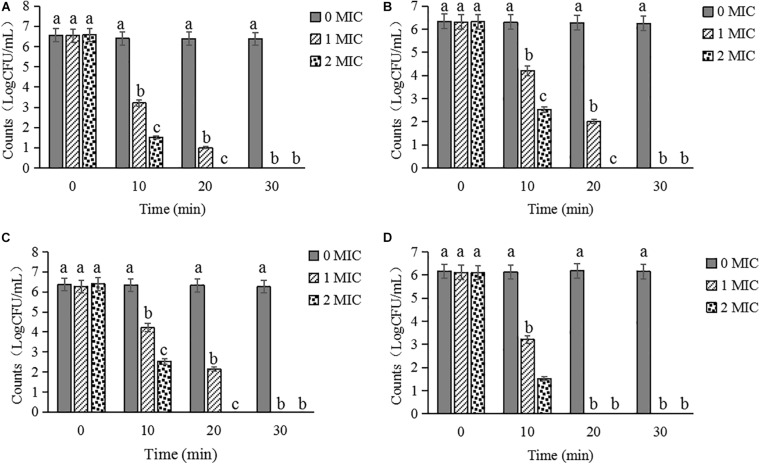
Inactivated effects of CBCE against *C. sakazakii* strains in biofilm on the **(A)** stainless steel tube, **(B)** tinplate, **(C)** glass, and **(D)** polystyrene. All strains on coupons were treated by 0, 1, and 2 MIC of CBCE for 30 min at 25°C. Bars represent the SD (*n* = 3). Different letters denote significant differences (*P* < 0.05).

The inactivation activities of CBCE against *C. sakazakii* ATCC 29544 in biofilm on stainless steel tube (Figure 8A), glass (Figure 8B), tinplate (Figure 8C), and polystyrene (Figure 8D) were evaluated. After treatments with CBCE, the number of viable bacteria in biofilm on the surfaces of the four materials was significantly reduced as compared to the control group (*P* < 0.05), and about 6.5 log CFU/mL of strains could be completely inactivated after a 1 MIC-30 min treatment or 2 MIC-20 min treatment. In addition, among the four materials, CBCE has the best antibacterial ability against *C. sakazakii* in biofilm on the polyetherone resin, followed by the stainless steel tube, tinplate, and glass.

## Discussion

So far, a variety of natural extracts, such as polyphenols, organic acids, anthocyanins, essential oils, and plant crude extracts, have been shown to have antibacterial effects against *C. sakazakii* (Amalaradjou et al., 2009; Li et al., 2016). Among these, plant crude extracts were more suitable as the natural disinfectants due to their low cost and simple processing technique. Yemis et al. (2019) found that the MIC of polyphenol-rich pomegranate peel extract against *C. sakazakii* was 20 mg/mL. Fei et al. (2020) explored the possibility of *Amaranthus tricolor* crude extract (ATCE) as a potential disinfectant and found that the MIC and MBC of ATCE against *C. sakazakii* were 20 and 40 mg/mL, respectively. In this study, MIC and MBC were 10 and 20 mg/mL, respectively, which indicated that CBCE had a better antibacterial effect against *C. sakazakii.*

ATP was closely related to the release, storage, and utilization of energy in the metabolism of substances, so it was considered as an important parameter that could reflect the survival status of cells (Guo et al., 2019). Our findings showed that the CBCE significantly reduced the intracellular ATP concentration of *C. sakazakii* cells, which is consistent with the changes of ATP concentration after olive oil polyphenol extract (OOPE), ATCE, citral, and syringic acid acted on *C. sakazakii* cells (Shi et al., 2016a,b; Fei et al., 2018a, 2020). Fei et al. (2018a) believed that the decrease of intracellular ATP concentration was related to the improvement of membrane permeability and excessive hydrolysis of ATP after treatment with natural extracts. Besides, some studies showed that the reduction of ATP synthase activity, loss of K^+^ inos, and increase in cell hydrolysis rate caused the decrease of ATP content in the cells treated by natural extracts (Pasqua et al., 2010; Sánchez et al., 2010; Khan et al., 2017).

Membrane potential, the potential difference between the two sides of the cell membrane in the resting state of the cell, is an important indicator to characterize the vitality of bacteria and play a pivotal role in the absorption of antibiotics and bactericidal action (Yun and Lee, 2017). When the cell is destroyed, the migration of Na^+^ can cause the cell membrane depolarization, while the leakage of K^+^ ions and changes of pH in cells can lead to the hyperpolarization (Bot and Prodan, 2009; Gries et al., 2013). In the current study, the cell membrane depolarization was found in the *C. sakazakii* strains after treatment with CBCE. Similar results have been reported in previous studies on the bacteriostatic process on nopal cactus methanolic extracts against *Vibrio cholerae*, lipoic acid against *C. sakazakii*, and ATCE against *Staphylococcus aureus* (Sánchez et al., 2010; Shi et al., 2016c; Guo L. et al., 2020). As a comparison, the cell membrane hyperpolarization was observed in the *C. sakazakii* cells treated by citral and syringic acid (Shi et al., 2016a,b).

Maintenance of pH_in_ homeostasis can ensure the normal operation of bacterial enzymatic reactions, DNA transcription, and protein synthesis (Bracey et al., 1998). In this study, after treatments with CBCE, the pH_in_ of the *C. sakazakii* cell dropped significantly, which suggested that the DNA and protein synthesis in the tested cell should be disrupted, leading to the growth of bacteria to be inhibited. Similarly, a significant pH_in_ reduction was also found in the tested bacteria during the inhibition of lipoic acid, ATCE, and citral against *C. sakazakii*, and mustard essential oil against *Escherichia coli* O157:H7 and *Salmonella typhi* (Turgis et al., 2009; Shi et al., 2016a,c; Fei et al., 2020). In addition, Fitzgerald et al. (2004) found that once the integrity of the cell membrane was destroyed, bacteria could not maintain a stable pH_in_ through ion exchange inside and outside of the cell membrane. Interestingly, the TEM results in our study showed that CBCE destroyed the cell morphology of *C. sakazakii*, which is consistent with the above idea.

Previous studies have taken the change in bacterial protein as one of the important ways to reveal the antibacterial mechanism of natural products and found that the bacterial protein of the tested bacteria significantly decreased after the treatments with natural products (Pasqua et al., 2010; Wang et al., 2015; Fei et al., 2018a; Guo L. et al., 2020). Chen et al. (2017) reported that the reduction of bacterial protein was associated with the increase of membrane permeability and obstruction in protein expression and synthesis. Similarly, after treatments with CBCE, the protein bands of *C. sakazakii* became much weaker, suggesting that the bacterial protein level decreased. In addition, we also found that the protein content in the supernatant increased significantly, which suggested that CBCE enhanced the membrane permeability of *C. sakazakii*, resulting in a large amount of intracellular protein leakage.

In this study, the amount of nucleic acid leaking from *C. sakazakii* cells treated by CBCE were significantly increased compared with untreated cells, which was consistent with the previous studies (Ulanowska et al., 2006; Sperotto et al., 2013; Cui et al., 2018). Ulanowska et al. (2006) found that the synthesis of DNA and RNA was disturbed when polyphenols entered the test cells, thereby reducing the expression of genetic information and resulting in antibacterial effects. Sperotto et al. (2013) reported that the natural products can lead to the decrease of the DNA gyrase and ribonucleic acid synthetase activity, thus hindering synthesis and restoration of nucleic acid substance in cell. Furthermore, the study reported by Cui et al. (2018) indicated that the reduction of DNA content under the action of natural products was associated with the destruction of cellular integrity and the damage to the original double helix structure of DNA.

A large number of researches indicated that one of the most important reasons that natural products effectively inhibit the food-borne pathogens is that they can cause the cellular morphology destruction and intracellular component leakage (Rhayour et al., 2003; Borges et al., 2013; Joshi et al., 2014; Fei et al., 2018a, 2020). In the current study, CBCE led to the irreversible changes of *C. sakazakii*, including cell deformation, collapse and damage, and cell fluid leakage, which was consistent with the previous findings of Fei et al. (2018a, 2020) who revealed the effects of OOPE and ATCE on the morphology of *C. sakazakii* cells. Meanwhile, the effects of natural products on the cell morphology of food-borne pathogenic bacteria are diverse, due to the differences in the antibacterial mechanism of natural products and the tolerance of the tested bacteria. Rhayour et al. (2003) found that after treatments with clove essential oil, the holes appeared on the cell surface of *E. coli*, while morphological changes occurred in *Bacillus subtilis*. Joshi et al. (2014) reported that blueberry proanthocyanidin can only lead to the appearance of pores and bubbles on the surface of *C. sakazakii* cells, resulting in the reversible antibacterial effect of blueberry proanthocyanidin.

It has been reported that *C. sakazakii* can attach to the surface of various materials to form the biofilm, thereby providing a certain physical protective barrier for the bacteria to resist a variety of external pressures, such as heat, osmotic pressure, disinfectants, and antibiotics (Borucki et al., 2003). At present, the common way to kill *C. sakazakii* on equipment and material surfaces in the food industry is to use the chemical disinfectants. However, the excessive and long-term use of chemical disinfectants was liable to cause drug dependence on bacteria to make it less effective, and the potential impact of its also cannot be ignored (Carlie et al., 2020). For example, sodium hypochlorite is so unfriendly to the environment that it has been banned in some European countries (Meireles et al., 2016). Meanwhile, as the common chemical disinfectants, benzalkonium chloride, oxyacetic acid, and chlorine dioxide had lower killing efficacy to *C. sakazakii* on the polystyrene surface and only removed 18% of the biofilm biomass (Torlak et al., 2015). In the present study, as a natural disinfectant, CBCE has the obvious bactericidal effects against *C. sakazakii* in biofilm on stainless steel tube, tinplate, glass, and polystyrene; among them, the antibacterial effect against this pathogen on the polystyrene surface was the greatest. Further, after treatments with 1 MIC of CBCE for 30 min at 25°C, approximately 6.5 log CFU/mL of *C. sakazakii* strains were fully inactivated, which was better than the antibacterial activity of coenzyme Q_0_ reported by Guo D. et al. (2020) who found that 1 MIC of coenzyme Q_0_ can significantly decrease but not completely inactivate the *C. sakazakii* strains on a stainless steel tube under the same treatment time and temperature.

## Conclusion

In summary, compared with the reported plant crude extracts, CBCE showed superior antibacterial activity against *C. sakazakii* and it exerts antibacterial effects by reducing the content of ATP, causing cell depolarization, reducing pH_in_, promoting the release of protein and nucleic acid substances, and destroying cell morphology. Moreover, CBCE can inactivate *C. sakazakii* in biofilm on processing equipment, packaging materials, and utensils. If only as a disinfectant, the safety of natural products and their effect on food sensory quality can be ignored; however, the dosage of CBCE needs to be optimized before application. In this study, we prepared the crude extracts of *C. buds*; therefore, CBCE should be purified in the following study in order to improve its antibacterial effect. In future studies, the focus of research may be on the determination of antibacterial components, combination of natural antibacterial substances and electrolytic water, and development of natural preservatives.

## Data Availability Statement

The original contributions presented in the study are included in the article/Supplementary Material, further inquiries can be directed to the corresponding author/s.

## Author Contributions

PF and YC conceived and designed the experiments. YC, MX, HF, XH, and MS performed the experiments. PF, YC, and HF supervised the project. YC, MX, XH, and LM analyzed the data. PF, YC, and MX wrote the manuscript. YW and BG contributed to the revision of the manuscript. All authors contributed to the article and approved the submitted version.

## Conflict of Interest

The authors declare that the research was conducted in the absence of any commercial or financial relationships that could be construed as a potential conflict of interest.

## Supplementary Material

Supplementary Figure 1 The images of *C. buds* and CBCE powder. **(A)** *C. buds* image, **(B)** CBCE powders image.
