# Supplementary figures and images for: Antibacterial Activity of Chrysanthemum buds Crude Extract Against Cronobacter sakazakii and Its Application as a Natural Disinfectant
Source: Front Microbiol. 2021 Feb 3;11:632177. doi: 10.3389/fmicb.2020.632177 (PMC7887297; doi:10.3389/fmicb.2020.632177)

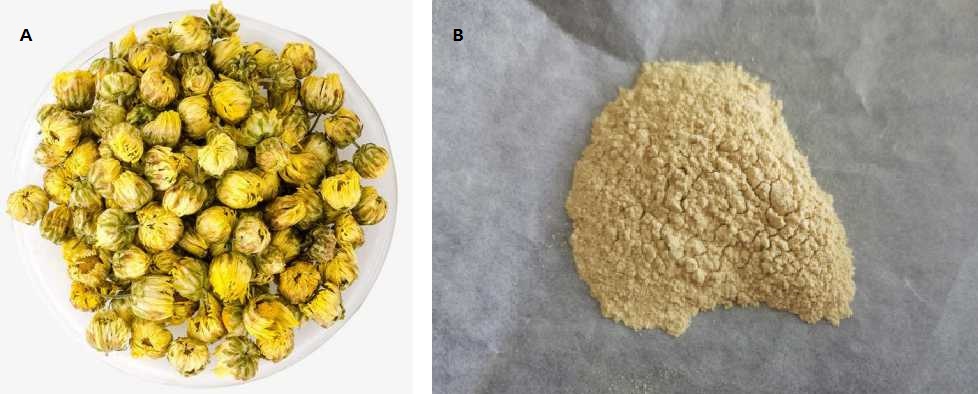

Supplement: Supplementary Figure 1 — The images of C. buds and CBCE powder. (A) C. buds image, (B) CBCE powders image. [file Image_1.jpeg]
