# Supplementary material for: Antibacterial Activity of Chrysanthemum buds Crude Extract Against Cronobacter sakazakii and Its Application as a Natural Disinfectant
Source: Front Microbiol. 2021 Feb 3;11:632177. doi: 10.3389/fmicb.2020.632177 (PMC7887297; doi:10.3389/fmicb.2020.632177)
Supplement: Supplementary file 2 [file Table_1.doc]

**Supplementary Table 1** The main chemical composition of CBCE used in this study.

| Composition | Content | Composition | Content |
| --- | --- | --- | --- |
| Total polyphenols | 30.4% | Camphor | 0.96% |
| Polysaccharide | 19.6% | Acacia-7-glucoside | 0.94% |
| Flavonoid | 13.2% | Lutedin | 0.87% |
| Moisture | 6.85% | Luteolin-7-glucoside | 0.85% |
| Protein | 2.38% | Borneol | 0.83% |
| Chlorogenic acid | 1.92% | Luteoloside | 0.72% |
| Fat | 1.03% | Acacetin | 0.74% |
| Quercetin | 1.26% | Baicalin | 0.65% |
| Linarin | 1.01% | Others | 15.79% |
